# Supplementary material for: Integrative QTL Mapping and Transcriptomic Profiling to Identify Growth-Associated QTL and Candidate Genes in Hong Kong Catfish (Clarias fuscus)
Source: Animals (Basel). 2025 Jun 9;15(12):1707. doi: 10.3390/ani15121707 (PMC12189263; doi:10.3390/ani15121707)
Supplement: Supplementary file 1 [file animals-15-01707-s001.zip › animals-3653486-supplementary.pdf]

Table S1. Quantitative real time PCR (qRT-PCR) primer sequences data.

Gene Name	Primer Sequences (5′-3′)
c9orf78	F: AGACAGGAGGCATCGTGGACAT<br>R: TTCTCGGGCAGTTCGTACAGGA
egfr	F: TGGCGGTGTCCTCTGTATCTCC<br>R: TTCTGCTCTGGTTGCTCGTTCG
mstn	F: GTATCCGCACACGCACTTGGT<br>R: TCACGAACAGCCACAGCGATC
eya4	F: CTCGCCTCCTCCTGACAGTGAT<br>R: TGACAGCCATCGGAGCATCCT
npm1	F: GTCAGGATGGCGTGCGTTGAT<br>R: TGCTGGCCGCTGATGTGAAC
β-actin	F: AGGCTGGATTCGCTGGAGATGAT<br>R: TGGTGACAATACCGTGCTCAATGG

Table S2. Phenotypic data on eight growth trait-related phenotypes from 200 offspring of *C. fuscus*.

Trait	Mean±SD	CV%
Body Weight (g)	2.22± 0.94	42.26
Body Length (cm)	5.39 ± 0.92	17.02
Body Height (cm)	0.89 ± 0.24	26.81
Body Width (cm)	0.95 ± 0.38	40.02
Caudal Peduncle Length (cm)	0.66 ± 0.16	24.31
Caudal Peduncle Height (cm)	0.39 ± 0.10	26.46
Orbital Diameter (cm)	0.25 ± 0.04	16.00
Pre-dorsal Length (cm)	1.93 ± 0.33	16.92

Table S3. 162 functional genes identified in the QTL interval for growth of *C.fuscus*.

Gene ID	Linkage Group	Gene Name
ID=gene-Cfus22195;	LG09	T-box transcription factor TBX2b
ID=gene-Cfus22196;	LG09	acetyl-CoA carboxylase 1
ID=gene-Cfus22197;	LG09	protein AATF isoform X2
ID=gene-Cfus22198;	LG09	LIM/homeobox protein Lhx1
ID=gene-Cfus08012;	LG17	retinoic acid receptor alpha-B isoform X2
ID=gene-Cfus08013;	LG17	antigen KI-67 isoform X1
ID=gene-Cfus08014;	LG17	speckle targeted PIP5K1A-regulated poly(A) polymerase isoform X2
ID=gene-Cfus08015;	LG17	peptide YY
ID=gene-Cfus08016;	LG17	MAGUK p55 subfamily member 2 isoform X4
ID=gene-Cfus08017;	LG17	SWI/SNF-related matrix-associated actin-dependent regulator of chromatin subfamily D member 2 isoform X2
ID=gene-Cfus08018;	LG17	SWI/SNF-related matrix-associated actin-dependent regulator of chromatin subfamily D member 2
ID=gene-Cfus08019;	LG17	ataxin-7protein 3
ID=gene-Cfus08020;	LG17	band 3 anion exchange protein
ID=gene-Cfus22377;	LG09	surfeit locus protein 1
ID=gene-Cfus22378;	LG09	60S ribosomal protein L7a
ID=gene-Cfus22379;	LG09	mediator of RNA polymerase II transcription subunit 22 isoform X2
ID=gene-Cfus22380;	LG09	uncharacterized protein C9orf78 homolog
ID=gene-Cfus22381;	LG09	ubiquitin carboxyl-terminal hydrolase 20 isoform X1
ID=gene-Cfus22382;	LG09	prostaglandin E synthase
ID=gene-Cfus22383;	LG09	mitochondrial import inner membrane translocase subunit Tim22
ID=gene-Cfus22384;	LG09	protein FAM57Bisoform X1
ID=gene-Cfus22385;	LG09	G8 domain-containing protein DDB_G0286311
ID=gene-Cfus22386;	LG09	ubiquitin carboxyl-terminal hydrolase 2isoform X2
ID=gene-Cfus22387;	LG09	ubiquitin carboxyl-terminal hydrolase 2isoform X1
ID=gene-Cfus22388;	LG09	alpha-tectorin
ID=gene-Cfus22389;	LG09	tubulin-specific chaperone cofactor Eprotein isoform X1
ID=gene-Cfus22390;	LG09	tubulin-specific chaperone cofactor Eprotein
ID=gene-Cfus22393;	LG09	uncharacterized protein si:ch211-215c18.3 isoform X2
ID=gene-Cfus22394;	LG09	uncharacterized protein LOC108260421
ID=gene-Cfus22395;	LG09	upstream stimulatory factor 1isoform X2
ID=gene-Cfus22396;	LG09	junctional adhesion molecule A isoform X1
ID=gene-Cfus22397;	LG09	ubiquitin-associated and SH3 domain-containing protein B
ID=gene-Cfus08020;	LG17	band 3 anion exchange protein
ID=gene-Cfus08021;	LG17	tumor necrosis factor receptor superfamily member 16
ID=gene-Cfus19401;	LG06	LOW QUALITY PROTEIN: uncharacterized protein LOC108410623, partial
ID=gene-Cfus20305;	LG07	kin of IRREprotein 3 isoform X4
ID=gene-Cfus04685;	LG13	SPARC-related modular calcium-binding protein 1 isoform X1

ID=gene-Cfus04686;	LG13	SPARC-related modular calcium-binding protein 1 isoform X3
ID=gene-Cfus04687;	LG13	coiled-coil domain-containing protein 177
ID=gene-Cfus04688;	LG13	pleckstrin homology domain-containing family D member 1
ID=gene-Cfus04689;	LG13	zinc transporter ZIP9 isoform X1
ID=gene-Cfus04690;	LG13	ALK tyrosine kinase receptor
ID=gene-Cfus04692;	LG13	leukocyte tyrosine kinase receptor
ID=gene-Cfus10886;	LG20	fibroblast growth factor 11
ID=gene-Cfus10887;	LG20	claudin-7
ID=gene-Cfus10888;	LG20	cornifelin homolog Bisoform X1
ID=gene-Cfus10889;	LG20	placenta-specific gene 8 protein
ID=gene-Cfus10890;	LG20	T-cell leukemia homeobox protein 3
ID=gene-Cfus10891;	LG20	nucleophosmin
ID=gene-Cfus10892;	LG20	Kv channel-interacting protein 1isoform X1
ID=gene-Cfus10893;	LG20	poly [ADP-ribose] polymerase 14isoform X2
ID=gene-Cfus10894;	LG20	histamine H2 receptor
ID=gene-Cfus10895;	LG20	soluble scavenger receptor cysteine-rich domain-containing protein SSC5D
ID=gene-Cfus10896;	LG20	complement C1qprotein 2
ID=gene-Cfus10897;	LG20	protocadherin gamma-C5isoform X4
ID=gene-Cfus10898;	LG20	protocadherin gamma-C5isoform X5
ID=gene-Cfus10899;	LG20	protocadherin gamma-C5isoform X8
ID=gene-Cfus10900;	LG20	protocadherin beta-16
ID=gene-Cfus10901;	LG20	protocadherin beta-16
ID=gene-Cfus10902;	LG20	protocadherin beta-16
ID=gene-Cfus10903;	LG20	uncharacterized protein LOC110504426
ID=gene-Cfus10904;	LG20	protocadherin alpha-2isoform X4
ID=gene-Cfus10905;	LG20	Protocadherin Alpha Subfamily C, 2
ID=gene-Cfus19780;	LG06	pyruvate dehydrogenase (acetyl-transferring) kinase isozyme 1, mitochondrial
ID=gene-Cfus19781;	LG06	neurabin-1isoform X3
ID=gene-Cfus05374;	LG14	arf-GAP with coiled-coil, ANK repeat and PH domain-containing protein 3isoform X2
ID=gene-Cfus05375;	LG14	tRNA pseudouridine synthase1 isoform X2
ID=gene-Cfus05376;	LG14	ATP-dependent RNA helicase DDX19A
ID=gene-Cfus05377;	LG14	calcium/calmodulin-dependent protein kinase II inhibitor 2
ID=gene-Cfus05378;	LG14	trypsin
ID=gene-Cfus05379;	LG14	trypsin
ID=gene-Cfus05380;	LG14	cytidine deaminase
ID=gene-Cfus05381;	LG14	dnaJ homolog subfamily C member 16
ID=gene-Cfus05382;	LG14	transmembrane and coiled-coil domains protein 1
ID=gene-Cfus05383;	LG14	mucin-5ACisoform X1
ID=gene-Cfus05384;	LG14	arginine-glutamic acid dipeptide repeats protein isoform X1
ID=gene-Cfus05385;	LG14	glutamine-rich protein 1
ID=gene-Cfus22359;	LG09	immediate early response gene 5protein

ID=gene-Cfus22360;	LG09	serine/threonine-protein phosphatase 2A activator
ID=gene-Cfus22361;	LG09	carnitine O-acetyltransferase isoform X2
ID=gene-Cfus22362;	LG09	FK506-binding protein 15 isoform X2
ID=gene-Cfus22363;	LG09	E3 ubiquitin-protein ligase LRSAM1
ID=gene-Cfus22364;	LG09	60S ribosomal protein L12
ID=gene-Cfus22365;	LG09	DNA polymerase epsilon subunit 3
ID=gene-Cfus22366;	LG09	protein CYR61
ID=gene-Cfus22367;	LG09	LIM domain transcription factor LMO4.1
ID=gene-Cfus22368;	LG09	tubulin beta-4B chain isoform X2
ID=gene-Cfus22369;	LG09	tubulin beta-4B chain isoform X1
ID=gene-Cfus22370;	LG09	Golgi-associated plant pathogenesis-related protein 1
ID=gene-Cfus08037;	LG17	protein MTO1 homolog
ID=gene-Cfus08038;	LG17	tectonic-3isoform X1
ID=gene-Cfus08039;	LG17	WD repeat-containing protein wdr-5.1isoform X1
ID=gene-Cfus08040;	LG17	sulfotransferase family cytosolic 1B member 1
ID=gene-Cfus08041;	LG17	sarcoplasmic/endoplasmic reticulum calcium ATPase 1
ID=gene-Cfus08042;	LG17	serine/threonine-protein phosphatase alpha-2 isoform
ID=gene-Cfus08043;	LG17	protein pelota homolog
ID=gene-Cfus08044;	LG17	progestin and adipoQ receptor family member 4
ID=gene-Cfus08045;	LG17	protein PAT1 homolog 2
ID=gene-Cfus08046;	LG17	transforming growth factor beta-1-induced transcript 1 protein isoform X1
ID=gene-Cfus08047;	LG17	transcription elongation factor b polypeptide 2
ID=gene-Cfus08048;	LG17	membrane-associated tyrosine- and threonine-specific cdc2-inhibitory kinase
ID=gene-Cfus08049;	LG17	zinc finger protein 629
ID=gene-Cfus08050;	LG17	mediator of RNA polymerase II transcription subunit 1
ID=gene-Cfus08052;	LG17	neurogenic differentiation factor 2
ID=gene-Cfus08053;	LG17	succinate receptor 1
ID=gene-Cfus08054;	LG17	protein phosphatase 1 regulatory subunit 1B isoform X1
ID=gene-Cfus08055;	LG17	uncharacterized protein LOC108273771 isoform X4
ID=gene-Cfus08057;	LG17	zinc finger BED domain-containing protein 1
ID=gene-Cfus08060;	LG17	macrophage mannose receptor 1
ID=gene-Cfus08061;	LG17	phenylethanolamine N-methyltransferase
ID=gene-Cfus08062;	LG17	zinc finger protein OZF
ID=gene-Cfus08063;	LG17	serine-rich coiled-coil domain-containing protein 2 isoform X1
ID=gene-Cfus08064;	LG17	glutamate receptor ionotropic, delta-1 isoform X2
ID=gene-Cfus08066;	LG17	glutamate receptor ionotropic, delta-1 isoform X1
ID=gene-Cfus08067;	LG17	glutamate receptor ionotropic, delta-1 isoform X2
ID=gene-Cfus08068;	LG17	hypothetical protein BRAFLDRAFT_63902
ID=gene-Cfus08779;	LG18	T-box transcription factor TBX18
ID=gene-Cfus08780;	LG18	T-box transcription factor TBX18
ID=gene-Cfus08781;	LG18	interleukin-1 receptor-associated kinase 1-binding protein 1
ID=gene-Cfus08782;	LG18	uncharacterized protein LOC108275973

ID=gene-Cfus08783;	LG18	PH-interacting protein
ID=gene-Cfus08784;	LG18	lebercilin
ID=gene-Cfus08785;	LG18	40S ribosomal protein S12
ID=gene-Cfus08786;	LG18	eyes absent homolog 4 isoform X2
ID=gene-Cfus22195;	LG09	T-box transcription factor TBX2b
ID=gene-Cfus22196;	LG09	acetyl-CoA carboxylase 1
ID=gene-Cfus22197;	LG09	protein AATF isoform X2
ID=gene-Cfus22198;	LG09	LIM/homeobox protein Lhx1
ID=gene-Cfus22199;	LG09	rRNA methyltransferase 1, mitochondrial
ID=gene-Cfus22200;	LG09	Meckel syndrome type 1 protein
ID=gene-Cfus20030;	LG06	tissue factor pathway inhibitor, partial
ID=gene-Cfus20031;	LG06	calcitonin gene-related peptide type 1 receptorisoform X1
ID=gene-Cfus20032;	LG06	uncharacterized protein LOC108266429
ID=gene-Cfus20033;	LG06	craniofacial development protein 2
ID=gene-Cfus20034;	LG06	signal transducer and activator of transcription 4 isoform X2
ID=gene-Cfus20035;	LG06	WD repeat-containing protein 75
ID=gene-Cfus20036;	LG06	solute carrier family 40 member 1
ID=gene-Cfus20037;	LG06	asparagine synthetase domain-containing protein 1
ID=gene-Cfus20038;	LG06	uncharacterized protein LOC108266707 isoform X1
ID=gene-Cfus20039;	LG06	ankyrin and armadillo repeat-containing protein
ID=gene-Cfus20040;	LG06	probable tRNA N6-adenosine threonylcarbamoyltransferase
ID=gene-Cfus20041;	LG06	probable inactive tRNA-specific adenosine deaminaseprotein 3
ID=gene-Cfus20042;	LG06	ORM1protein 1
ID=gene-Cfus20043;	LG06	PMS1 protein homolog 1
ID=gene-Cfus20044;	LG06	myostatin
ID=gene-Cfus20045;	LG06	small membrane A-kinase anchor protein
ID=gene-Cfus20046;	LG06	3-hydroxyisobutyryl-CoA hydrolase, mitochondrial
ID=gene-Cfus20047;	LG06	GDP-fucose protein O-fucosyltransferase 2 isoform X2
ID=gene-Cfus20048;	LG06	ATP-binding cassette sub-family A member 12
ID=gene-Cfus20049;	LG06	BRCA1-associated RING domain protein 1
ID=gene-Cfus20050;	LG06	von Willebrand factor C domain-containing protein 2
ID=gene-Cfus20051;	LG06	complement C1q tumor necrosis factor-related protein 3
ID=gene-Cfus20052;	LG06	IKAROS Family Zinc Finger 2
ID=gene-Cfus20053;	LG06	IKAROS Family Zinc Finger 2
ID=gene-Cfus20054;	LG06	craniofacial development protein 2-like, partial
ID=gene-Cfus20058;	LG06	Epidermal Growth Factor Receptor (receptor tyrosine-protein kinase erbB-4)
ID=gene-Cfus20059;	LG06	Epidermal Growth Factor Receptor
ID=gene-Cfus20061;	LG06	Epidermal Growth Factor Receptor
ID=gene-Cfus20062;	LG06	carbamoyl-phosphate synthase
ID=gene-Cfus20063;	LG06	myosin light chain 1, skeletal muscle isoform
ID=gene-Cfus20064;	LG06	microtubule-associated protein 2 isoform X1
ID=gene-Cfus20065;	LG06	microtubule-associated protein 2 isoform X16

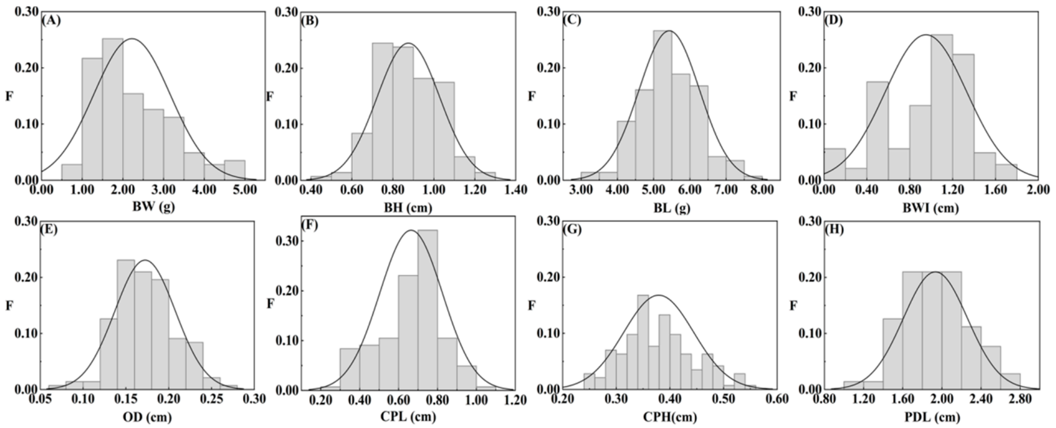

Figure S1. Frequency distribution of growth traits in the F1 full-sibling population (N = 200): (**A**) body weight, (**B**) body height, (**C**) body length, (**D**) body width, (**E**) orbital diameter, (**F**) caudal peduncle length, (**G**) caudal peduncle height, and (**H**) pre-dorsal length. The vertical axis represents the percentage of individuals within each interval relative to the total population.
